# Randomized, Sham-Controlled, Double-Blind Trial of the Intibia System in Urge Urinary Incontinence: Quality-of-Life Outcomes

**DOI:** 10.1007/s00192-026-06636-1

**Published:** 2026-06-22

**Authors:** Jeffrey Mangel, Kevin Miller, Ty Erickson, Alexis Dieter, Jan-Paul Roovers, Catherine A. Matthews

**Affiliations:** 1 Division of Urogynecology and Reconstructive Pelvic Surgery, MetroHealth Medical Center, Case Western Reserve University School of Medicine, MetroHealth Medical Center, 2500 MetroHealth Drive, Cleveland, OH 44109 USA; 2 School of Medicine-Wichita Department of Obstetrics & Gynecology, University of Kansas, Wichita, KS USA; 3 Division of Urogynecology, Kirk Kevorkian School of Medicine, Las Vegas, NV USA; 4 Urogynecology & Reconstructive Pelvic Surgery, MedStar Washington Hospital Center, Departments of OBGYN and Urology, Georgetown University School of Medicine, Washington, DC USA; 5 Department of Obstetrics and Gynaecology, Amsterdam Reproduction and Development Research Institute, Amsterdam UMC, University of Amsterdam, Amsterdam, The Netherlands; 6 Division Head and Fellowship Director, Female Pelvic Health; Department of Urology, Wake Forest University School of Medicine, Winston-Salem, NC USA

**Keywords:** Intibia, Implantable tibial nerve stimulation, Quality of life, Randomized, Sham

## Abstract

**Introduction and Hypothesis:**

Urgency urinary incontinence (UUI) is associated with significant impairments to quality of life (QoL) and overall social well-being. The aim of this analysis was to assess the impact of the Intibia System implantable tibial nerve stimulation (ITNS) device on QoL in patients with UUI and compare with a sham control.

**Methods:**

The Intibia Pivotal Study was a prospective, randomized, sham-controlled, double-blind trial across the USA and the Netherlands. A total of 208 subjects were randomized to either an ITNS treatment group (ITNS-ON, *n* = 139) or a sham control group (ITNS-SHAM, *n* = 69). ITNS-SHAM subjects crossed over after 3 months. Disease-specific questionnaires regarding QoL and patient-reported outcomes were used to assess the impact of the Intibia System at 3, 6, and 12 months post-implant.

**Results:**

At 3 months, significantly more ITNS-ON subjects reported feeling “much better” or “very much better” than ITNS-SHAM subjects (29.2% vs 13.4%, *p* = 0.0133); ITNS-ON subjects also exceeded the minimally clinically important difference (MCID) at a higher rate than the ITNS-SHAM group for the Overactive Bladder Questionnaire Short Form Symptom Bother (62.8% vs 41.8%; *p* = 0.0046) and Health-Related Quality of Life sub-scales (57.4% vs 42.4%; *p* = 0.0463). At 12 months, 83% of ITNS-ON subjects reported any level of improvement compared with baseline. Significant improvements within the ITNS-ON group were observed in the International Consultation on Incontinence Questionnaire – Urinary Incontinence Short Form over 12 months, with 71.5% of subjects exceeding the MCID.

**Conclusions:**

The Intibia System demonstrated statistically and clinically meaningful improvements in self-reported, disease-specific QoL measures in patients with UUI compared with sham at 3 months and compared with baseline at 12 months.

## Introduction

Overactive bladder (OAB) is characterized by urinary urgency with or without incontinence, nocturia, or urinary frequency [1]. OAB increases with age and worldwide prevalence rates range from 9% to 43% in women and 7% to 27% in men [2]. Urge urinary incontinence (UUI) affects approximately one third of patients with OAB and is reported to be a significantly bothersome symptom [3, 4]. UUI is associated with substantial comorbidities including increased body mass index (BMI), diabetes, falls in the elderly, and death [4]. Loss of bladder control in patients with UUI is also associated with a marked reduction in quality of life (QoL), affecting physical, mental and social well-being [4]. Urinary incontinence is often stigmatized, leading to physical and psychosocial restrictions. OAB patients with incontinence specifically report the highest impairments in work productivity [5], and UUI has been shown to be a significant risk factor for anxiety and depression [4, 6].

Management of OAB symptoms usually starts with conservative approaches such as behavioral modification, pelvic floor muscle training, and/or pharmacological therapies. After failure of more conservative treatment options for UUI, another treatment option includes neuromodulation, delivered either centrally via sacral nerve stimulation (SNS) or peripherally via percutaneous tibial nerve stimulation (PTNS) or implantable tibial nerve stimulation (ITNS). Stimulation of the tibial nerve sends impulses to the sacral nerve plexus in the spinal cord, innervating the bladder. PTNS offers significant UUI relief and improvements in QoL, but requires repeated in-office visits, which contributes to poor patient compliance [7, 8]. In recent years, ITNS devices have been developed as an alternative strategy, allowing patients to achieve symptom management while at home. Published trials utilizing ITNS devices have shown considerable QoL improvements in patients with UUI [9–11], but were not sham controlled. This is remiss as many OAB treatments have a high rate of placebo effect [12, 13].

The Intibia System is an ITNS device with a nonrechargeable battery and an integrated electrode lead designed to provide stimulation adjacent to the tibial nerve. Here we report the planned secondary analysis of the QoL outcomes from the Intibia Pivotal Study: a double-blind, randomized, sham-controlled trial. The analyses presented here evaluated the impact of the Intibia System over a sham-control group using multiple patient-reported outcomes, including QoL measures.

## Materials and Methods

### Trial Design

This is a planned secondary analysis of the QoL additional outcomes of subjects enrolled in the Intibia Pivotal Study, a prospective, randomized, sham-controlled, double-blind trial that included sites from the USA and the Netherlands (Clinicaltrials.gov, NCT05250908). The primary study analysis will be reported elsewhere. Enrollment ran from March 2022 to May 2024. A total of 23 sites performed device implantation (21 in the USA and 2 in the Netherlands). Prior to subject enrolment at each site, the study was approved by the appropriate central or local Institutional Review Board (IRB)/Research Ethics Committees and adhered to standards of good clinical practice. The Western Copernicus Group provided study-wide and clinical research site IRB oversight, whereas a number of local IRBs retained oversight of clinical sites as applicable per local institutional policies and procedures. Written informed consent was collected from all participants prior to commencement of any study procedures. Subjects were randomized based on randomly ordered permuted blocks and stratified by site and gender. Subjects were assigned to one of two groups in a 2:1 ratio: an ITNS treatment group (ITNS-ON) or a sham control group (ITNS-SHAM). Study subjects, investigators, and study staff were blinded to study group assignment until after assessments were completed at the 3-month visit. All randomized subjects were implanted with the Intibia System and the device was left off for 2 weeks to allow for recovery. After 2 weeks, subjects were programmed for stimulation and either activated (ITNS-ON) or left off (ITNS-SHAM) until the 3-month visit, when all subjects were unblinded after completion of bladder diaries and questionnaires. ITNS-SHAM subjects were crossed over after the 3-month visit and interactive stimulation (ability to adjust stimulation levels based on subject feedback) commenced. Follow-up visits occurred at 2 weeks and 3, 6, and 12 months post-implant.

### Study Population

The study included adult men and women (aged 22–80 years) with a minimum of four UUI episodes on a 72-h voiding diary, and at least one UUI episode per day. More conservative and/or behavioral therapies, including pharmacological treatments, bladder/sphincter training, timed voiding, behavioral modifications, fluid management, pelvic floor muscle strengthening, and/or pad usage, had failed in subjects included in the trial. Subjects abstained from OAB medications for the duration of the 12-month follow-up. All other medications that could impact bladder function required a stable dose for at least 4 weeks prior to baseline voiding diary and at least 4 weeks prior to any follow-up visit. Urodynamic testing was not performed on any of the study patients. The diagnosis of OAB was made on the basis of questionnaire responses and negative cough stress test. Key exclusion criteria were a diagnosis of stress or mixed urinary incontinence, post-void residual urine volume > 30% of total voided volume, any prior treatment with nerve stimulation (e.g., PTNS, SNS), treatment with botulinum toxin therapy within 6 months, uncontrolled diabetes mellitus, chronic pain, or subjects with any neurological condition that could interfere with normal bladder or tibial nerve function (stroke, epilepsy, multiple sclerosis, spinal cord injury, etc.).

### Implant Procedure and Device Settings

The Intibia System is an electronic tibial nerve stimulator comprising a miniaturized pulse generator and integrated electrode lead powered by a nonrechargeable lithium cell. During surgery, the pulse generator was implanted subcutaneously above the fascia whereas the integrated lead was inserted and deployed into the sub-fascial space adjacent to the tibial nerve, all via one small incision. A programming set comprising a tablet and wand was used during the procedure to deliver stimulation and confirm lead location. Subjects were allowed 2 weeks for recovery until the first postoperative visit when the device was activated, and programming settings were established. At 2 weeks, amplitudes were set for all subjects based on sensory or visible motor feedback (flexion of the great toe) and the subject’s comfort level. Subjects were told the following information at the 2-week visit: “Your device will be programmed to therapy that may or may not be effective. Whether the therapy is effective or not, you may or may not feel sensation.” The ITNS-SHAM group were not activated. For the ITNS-ON group, stimulation was applied at the established amplitude threshold at a frequency of 20 Hz with a 200-μs pulse duration. Once activated, stimulation was turned on for 30 min daily for 2 weeks, followed by 30 min of weekly stimulation at the subject’s preferred time for the remainder of the study.

### Outcomes Evaluated

This planned analysis of the additional outcomes related to QoL from the Intibia Pivotal Study included multiple validated assessments of patient-reported changes to QoL and overall satisfaction. The Patient Global Impression of Improvement (PGI-I) questionnaire was first administered to assess patient perceived improvement in the bladder condition following device implantation. For OAB-specific symptoms the Overactive Bladder Questionnaire Short Form (OABq-SF), comprising two sub-scales to assess changes in symptom bother and health-related quality of life (HRQL) was administered. A score change > 10 exceeds the minimally clinically important difference (MCID) for both OABq-SF subscales [14]. Changes in patient-reported impact of urinary incontinence were assessed using the International Consultation on Incontinence Questionnaire - Urinary Incontinence Short Form (ICIQ-UI SF). Three-day voiding diaries were used to assess the number of baseline UUI episodes.

### Statistical Analyses

The Intibia Pivotal Study was designed to randomize up to 225 subjects under a 2:1 randomization scheme to provide at least 90% power to test both the primary (percentage of subjects achieving ≥50% reduction in UUI episodes at 3 months and 12 months relative to baseline) and secondary outcomes of the study. All statistical analyses were performed by an independent statistician using the SAS System software, Version 9.4 or above. Comparisons between groups at 3 months, and within groups relative to baseline were made using appropriate tests denoted within each table below. The intent-to-treat (ITT) population consisted of all randomized subjects analyzed according to their randomization assignment. All data presented in these analyses were conducted on the ITT population with complete data sets only. The respective sample size is indicated within each table/figure.

## Results

**Fig. 1:**
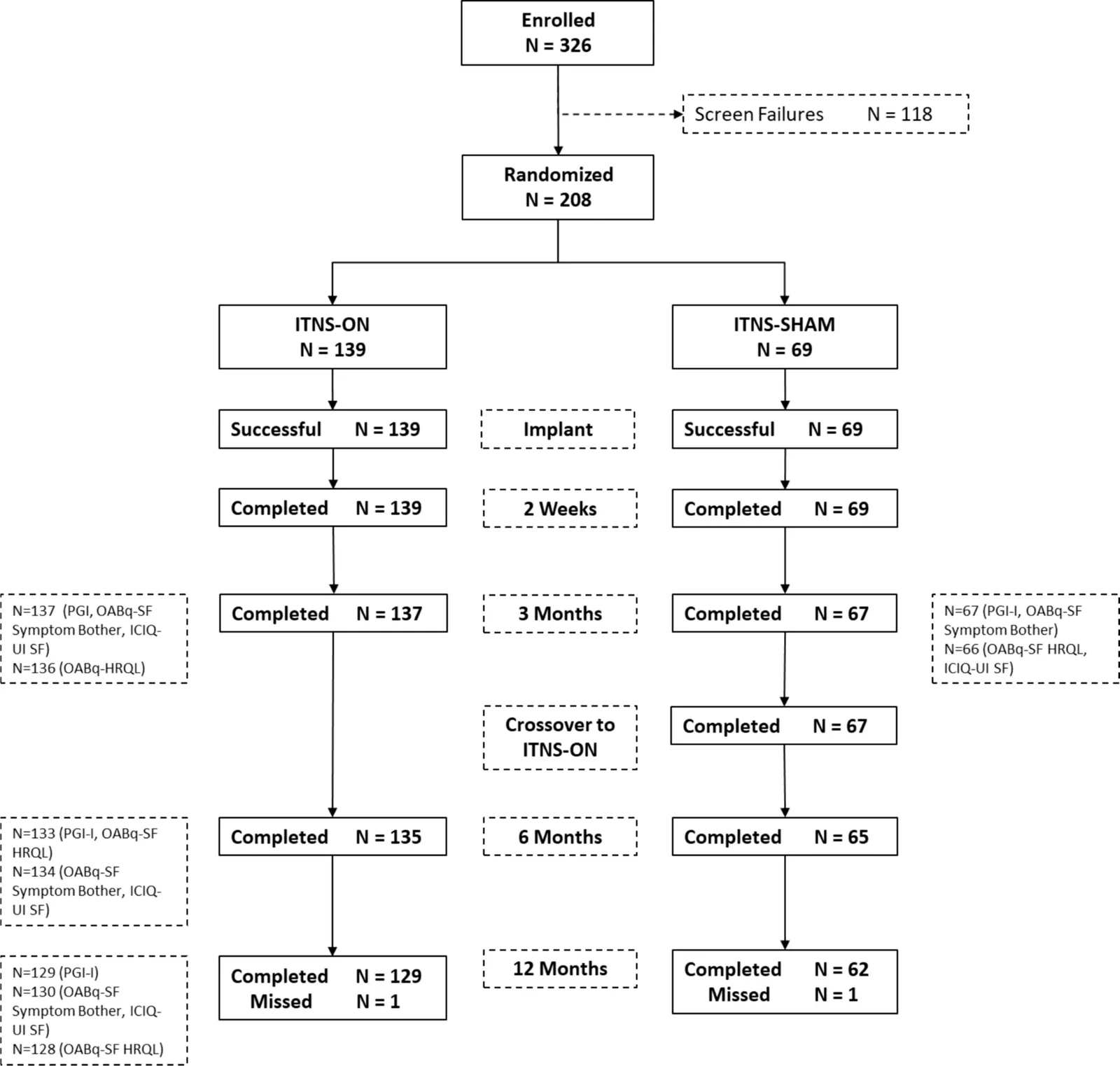
Subject disposition. *HRQL* health-related quality of life, *ICIQ-UI SF* International Consultation on Incontinence Questionnaire – Urinary Incontinence Short Form, *ITNS* implantable tibial nerve stimulation, *OABq-SF* Overactive Bladder Questionnaire Short Form, *PGI-I* Patient Global Impression of Improvement

A total of 326 subjects were enrolled, and 208 subjects were randomized to either the ITNS-ON group (*n* = 139) or the ITNS-SHAM group (*n* = 69). All randomized subjects were implanted and completed the 2-week visit. A total of 137 (98.6%) ITNS-ON and 67 (97.1%) ITNS-SHAM subjects completed the 3-month visit and a total of 129 (92.8%) ITNS-ON subjects and 62 (89.6%) ITNS-SHAM subjects completed the 12-month visit. Withdrawal occurred owing to adverse events (*n* = 7), perceived loss of efficacy (*n* = 4), voluntary withdrawal (*n* = 3), and lost to follow-up (*n* = 1), and 2 subjects missed the 12-month visit (Consort Diagram, Fig. 1).

Subject demographics and baseline characteristics are shown in Table 1. Subjects were predominantly female (98.6%) with a median age of 66 years for both groups (range 32–80 for ITNS-ON and 37–79 for ITNS-SHAM). All subjects presented with UUI symptoms (100%) and the majority also presented with symptoms of urgency (88.9%), frequency (78.8%), and nocturia (70.2%). Overall, subjects reported significant baseline UUI symptoms with similar average UUI episodes per day of 4.30 ± 2.73 in the ITNS-ON group and 4.52 ± 2.88 in the ITNS-SHAM group (*p* = 0.5927 between groups). Subjects reported a median duration of OAB symptoms of 7.16 years in the ITNS-ON group and 5.16 years in the ITNS-SHAM group (*p* = 0.2593 between groups).

**Table 1:** Demographics and baseline characteristics

	ITNS-ON (*N* = 139)	ITNS-SHAM (*N* = 69)	*p* value*
Age, mean ± SD	63.9 ± 10.3	64.7 ± 8.9	0.6053**
BMI, mean ± SD	32.2 ± 7.5	31.6 ± 7.1	0.5428**
Sex, *n*/*N* (%)			
Female	137/139 (98.6)	68/69 (98.6)	0.9953***
Male	2/139 (1.4)	1/69 (1.4%)	
Ethnicity (*n*/*N* (%))			
Hispanic or Latino	8/139 (5.8)	5/69 (7.2)	0.6351****
Not Hispanic or Latino	127/139 (91.4)	60/69 (87.0)	
Not disclosed	4/139 (2.9)	4/69 (5.8)	
Race (not mutually exclusive, *n*/*N* (%))			
American Indian or Alaska Native	0/139 (0.0)	1/69 (1.4)	0.0016*****
Asian	0/139 (0.0)	1/69 (1.4)	
Black or African American	6/139 (4.3)	8/69 (11.6)	
White	133/139 (95.7)	57/69 (82.6)	
Other	0/139 (0.0)	2/69 (2.9)	
Symptoms of overactive bladder, *n*/*N* (%)			
Urge urinary incontinence	139/139 (100.0)	69/69 (100.0)	N/A
Urgency	124/139 (89.2)	61/69 (88.4)	0.8620***
Frequency	110/139 (79.1)	54/69 (78.3)	0.8842***
Nocturia	96/139 (69.1)	50/69 (72.5)	0.6138***
Mean UUI episodes per day ± SD (*N*)	4.30 ± 2.73 (138)	4.52 ± 2.88 (69)	0.5927**

*BMI* body mass index, *ITNS* implantable tibial nerve stimulation, *N/A* not applicable, *SD* standard deviation

*Compares ITNS-ON and ITNS-SHAM groups

**Two-sample *t* test

***Chi-squared test

****Chi-squared test, excluding subjects who chose not to disclose their ethnicity

*****Chi-squared test, comparing subjects who reported white only versus all other races, excluding subjects who chose not to disclose their race

After 3 months, a significantly higher proportion of ITNS-ON subjects reported feeling “much better” or “very much better” compared with the ITNS-SHAM group (29.2% ITNS-ON vs 13.4% ITNS-SHAM; *p* = 0.0133) as measured by the PGI-I. By 12 months, 83% of ITNS-ON subjects reported any level of improvement, and over half (53.5%) reported feeling “much better” or “very much better” (Table 2).

**Table 2:** Patient Global Impression of Improvement (PGI-I)

	ITNS-ON: 3 months (*N* = 137)	ITNS-SHAM: 3 months (*N* = 67)	ITNS-ON: 6 months (*N *= 135)	ITNS-ON: 12 months (*N *= 129)
PGI-I score, *n*/*N* (%)				
Very much better	11/137 (8.0)	2/67 (3.0)	27/133 (20.3)	18/129 (14.0)
Much better	29/137 (21.2)	7/67 (10.4)	39/133 (29.3)	51/129 (39.5)
A little better	39/137 (28.5)	12/67 (17.9)	44/133 (33.1)	38/129 (29.5)
No change	52/137 (38.0)	38/67 (56.7)	20/133 (15.0)	13/129 (10.1)
A little worse	4/137 (2.9)	2/67 (3.0)	1/133 (0.8)	5/129 (3.9)
Much worse	0/137 (0.0)	5/67 (7.5)	2/133 (1.5)	2/129 (1.6)
Very much worse	2/137 (1.5)	1/67 (1.5)	0/133 (0.0)	2/129 (1.6)
Much better or very much better				
*n*/*N* (%)	40/137 (29.2)	9/67 (13.4)	66/133 (49.6)	69/129 (53.5)
95% confidence limits for proportion	21.6%, 36.8%	5.3%, 21.6%	41.1%, 58.1%	44.9%, 62.1%
Difference in proportions (95% CI)	–	15.8% (4.6%, 26.9%)	–	–
*p* value*	–	0.0133	–	–

*CI* confidence interval, *ITNS* implantable tibial nerve stimulation

*p value compares ITNS-ON with ITNS-SHAM at 3 months using a normal approximation (*z* test) for difference in proportions

The OABq-SF consists of Symptom Bother and HRQL sub-sections. At 3 months relative to baseline, ITNS-ON subjects reported significant improvements over ITNS-SHAM subjects in both the OABq-SF Symptom Bother Score (−18.5 ± 20.4 ITNS-ON vs −10.2 ± 19.9 ITNS-SHAM; *p* = 0.0068) and the OABq-SF HRQL total score (17.2 ± 20.2 ITNS-ON vs 7.2 ± 18.2 ITNS-SHAM; *p* = 0.0007). A significantly higher proportion of ITNS-ON subjects than ITNS-SHAM subjects exceeded the MCID for improvement in the Symptom Bother (62.8% ITNS-ON vs 41.8% ITNS-SHAM; *p* = 0.0046) and HRQL scales (57.4% ITNS-ON vs 42.4% ITNS-SHAM; *p* = 0.0463) at 3 months relative to baseline (Tables 3, 4).

**Table 3:** Overactive Bladder Questionnaire Short Form (OABq-SF) Symptom Bother scores

	ITNS-ON: baseline (*N *= 139)	ITNS-SHAM: baseline (*N* = 69)	ITNS-ON: 3 months (*N* = 137)	ITNS-SHAM: 3 months (*N* = 67)	ITNS-ON: 6 months (*N* = 135)	ITNS-ON: 12 months (*N* = 129)
OABq-SF Symptom Bother Score						
*N*	139	69	137	67	134	130
Mean ± SD	64.3 ± 17.1	61.4 ± 18.7	45.9 ± 22.4	51.0 ± 21.6	39.4 ± 22.9	38.3 ± 21.5
Median	63.3	60.0	43.3	46.7	36.7	36.7
Q1, Q3	53.3, 76.7	46.7, 76.7	26.7, 63.3	33.3, 66.7	23.3, 53.3	20.0, 50.0
Minimum, maximum	23.3, 100.0	16.7, 100.0	6.7, 100.0	16.7, 100.0	0.0, 100.0	0.0, 96.7
Change from baseline in OABq-SF Symptom Bother Score						
*N*	–	–	137	67	134	130
Mean ± SD	–	–	−18.5 ± 20.4	−10.2 ± 19.9	−24.9 ± 21.2	−25.6 ± 21.2
Median	–	–	−20.0	−6.7	−25.0	−30.0
Q1, Q3	–	–	−30.0, −3.3	−23.3, 0.0	−40.0, −10.0	−40.0, −10.0
Minimum, maximum	–	–	−66.7, 40.0	−56.7, 50.0	−70.0, 30.0	−73.3, 33.3
Difference between means (95% CI)	–	–	–	−8.26 (−14.21, −2.30)****	–	–
*p* value*	–	–	–	0.0068*****	–	–
*p* value** for change from baseline	–	–	< 0.001***	< 0.001******	< 0.001******	< 0.001***
Change from baseline in OABq-SF Symptom Bother Score MCID						
< −10	–	–	86/137 (62.8)	28/67 (41.8)	99/134 (73.9)	93/130 (71.5)
≥ −10	–	–	51/137 (37.2)	39/67 (58.2)	35/134 (26.1)	37/130 (28.5)
*p* value*	–	–	–	0.0046*******	–	–

Symptom Bother score is calculated as the sum of the six questionnaire items, each ranging from 1 (not at all) to 6 (a very great deal). The raw score is transformed to a normalized score that ranges from 0 to 100, with higher scores indicative of greater symptom bother

*CI* confidence interval, *MCID* minimally clinically important difference, *SD* standard deviation

*Compares ITNS-SHAM with ITNS-ON at 3 months

**Compares respective visit with baseline within the same randomization arm

***Wilcoxon signed-rank test

****Difference between means and 95% confidence limits are for two-sample *t* test

*****Two-sample *t* test

******Paired *t* test

*******Chi-squared test

**Table 4:** Overactive Bladder Questionnaire Short Form (OABq-SF) health-related quality of life (HRQL) total scores

	ITNS-ON: baseline (*N* = 139)	ITNS-SHAM: baseline (*N* = 69)	ITNS-ON: 3 months (*N* = 137)	ITNS-SHAM: 3 months (*N* = 67)	ITNS-ON: 6 months (*N* = 135)	ITNS-ON: 12 months (*N* = 129)
OABq-SF HRQL score						
*N*	138	69	137	66	134	129
Mean ± SD	49.1 ± 19.5	51.5 ± 21.7	66.2 ± 24.6	59.6 ± 22.5	74.4 ± 22.4	74.0 ± 22.6
Median	48.5	55.4	70.8	63.1	81.5	81.5
Q1, Q3	35.4, 61.5	35.4, 64.6	47.7, 89.2	47.7, 75.4	58.5, 92.3	60.0, 92.3
Minimum, maximum	6.2, 95.4	3.1, 95.4	6.2, 100.0	9.2, 100.0	7.7, 100.0	1.5, 100.0
Change from baseline in OABq-SF HRQL score						
*N*	–	–	136	66	133	128
Mean ± SD	–	–	17.2 ± 20.2	7.2 ± 18.2	25.3 ± 20.7	24.6 ± 20.3
Median	–	–	15.4	3.1	24.6	23.1
Q1, Q3	–	–	2.3, 30.0	−6.2, 20.0	10.8, 40.0	9.2, 39.2
Minimum, maximum	–	–	−26.2, 81.5	−49.2, 49.2	−21.5, 81.5	−16.9, 73.8
Difference between means (95% CI)	–	–	–	10.09 (4.31, 15.88)****	–	–
*p* value*	–	–	–	0.0007*****	–	–
*p* value** for change from baseline	–	–	< 0.001***	0.0021******	< 0.001******	< 0.001******
Change from baseline in OABq-SF HRQL score MCID						
> 10	–	–	78/136 (57.4)	28/66 (42.4)	101/133 (75.9)	95/128 (74.2)
≤ 10	–	–	58/136 (42.6)	38/66 (57.6)	32/133 (24.1)	33/128 (25.8)
*p* value*	–	–	–	0.0463*******	–	–

HRQL score is calculated as the sum of the 13 questionnaire items, each ranging from 1 (none of the time) to 6 (all of the time). The raw score is transformed to a normalized score that ranges from 0 to 100, with higher scores indicative of better HRQL

*CI* confidence interval, *MCID* minimally clinically important difference, *SD* standard deviation

*Compares ITNS-SHAM with ITNS-ON at 3 months

**Compares respective visit to baseline within the same randomization arm

***Wilcoxon signed-rank test

****Difference between means and 95% confidence limits are for two-sample *t* test

*****Two-sample *t* test

******Paired *t* test

*******Chi-squared test

Within the ITNS-ON group, the percentage of ITNS-ON subjects exceeding the MCID at 12 months relative to baseline also improved to 71.5% for the OABq-SF Symptom Bother and 74.2% for the OABq-SF HRQL Total Scores (Tables 3, 4). OABq-SF Symptom Bother scores were significantly reduced by an average of 25.6 ± 21.2 points relative to baseline (*p* < 0.001) and OABq-SF HRQL total scores significantly improved by an average of 24.6 ± 20.3 points (*p* < 0.001).

Both ITNS-ON and ITNS-SHAM groups reported significant improvements in the International Consultation on Incontinence Questionnaire – Urinary Incontinence Short Form (ICIQ-UI SF) total score at 3 months relative to baseline, with no significant differences between groups (−2.5 ± 4.2 ITNS-ON vs −1.9 ± 3.8 ITNS-SHAM; *p* = 0.3687 between groups). A score change greater than 1.74 exceeds the MCID for the ICIQ-UI SF [15]. Within the ITNS-ON group, 68.7% and 71.5% of subjects reported an improvement exceeding the ICIQ-UI SF MCID after 6 and 12 months, respectively (Table 5).

**Table 5:** International Consultation on Incontinence Questionnaire – Urinary Incontinence Short Form (ICIQ-UI SF) total scores

	ITNS-ON: baseline (*N* = 139)	ITNS-SHAM: baseline (*N* = 69)	ITNS-ON: 3 months (*N* = 137)	ITNS-SHAM: 3 months (*N* = 67)	ITNS-ON: 6 months (*N* = 135)	ITNS-ON: 12 months (*N* = 129)
ICIQ-UI SF total score						
*N*	139	69	137	66	134	130
Mean ± SD	14.3 ± 3.3	14.4 ± 3.2	11.8 ± 4.7	12.5 ± 4.2	9.8 ± 5.0	9.6 ± 5.0
Median	15.0	15.0	12.0	12.0	10.0	9.0
Q1, Q3	12.0, 17.0	13.0, 17.0	8.0, 15.0	9.0, 16.0	5.0, 14.0	5.0, 13.0
Minimum, maximum	4.0, 21.0	3.0, 20.0	3.0, 20.0	3.0, 21.0	0.0, 20.0	0.0, 21.0
Change from baseline in ICIQ-UI SF total score						
*N*	–	–	137	66	134	130
Mean ± SD	–	–	−2.5 ± 4.2	−1.9 ± 3.8	−4.4 ± 4.9	−4.5 ± 4.9
Median	–	–	−2.0	−2.0	−5.0	−5.0
Q1, Q3	–	–	−5.0, 1.0	−5.0, 1.0	−8.0, −1.0	−7.0, −1.0
Minimum, maximum	–	–	−16.0, 8.0	−11.0, 7.0	−17.0, 6.0	−17.0, 10.0
Difference between means (95% CI)	–	–	–	−0.55 (−1.75, 0.65)****	–	–
*p* value*	–	–	–	0.3687**	–	–
*p* value** for change from baseline	–	–	< 0.001***	< 0.001***	< 0.001***	< 0.001***
Change from baseline in ICIQ-UI SF score MCID						
< −1.74	–	–	80/137 (58.4)	34/66 (51.5)	92/134 (68.7)	93/130 (71.5)
≥ −1.74	–	–	57/137 (41.6)	32/66 (48.5)	42/134 (31.3)	37/130 (28.5)
*p* value*	–	–	–	0.3548*****	–	–

ICIQ-UI total score is calculated as the sum of the three questionnaire items. The total score ranges from 0 to 21, with higher scores indicating greater severity and impact of urinary incontinence

*CI* confidence interval, *MCID* minimally clinically important difference, *SD* standard deviation

*Compares ITNS-SHAM with ITNS-ON at 3 months

**Compares respective visit with baseline within the same randomization arm

***Wilcoxon signed-rank test

****Difference between means and 95% confidence limits are for two-sample *t* test

*****Chi-squared test

## Discussion

This analysis of the quality-of-life outcomes from the Intibia Pivotal Study demonstrated that there were significantly greater improvements in QoL measures in the active ITNS stimulation group than in the sham controls, and within the ITNS-ON group at 12 months than at baseline. This study provides critical therapeutic information that was missing from other ITNS studies that did not include a placebo control group [9–11], which is relevant given previous significant placebo responses to OAB interventions [12, 13]. Given the critical need for a randomized, sham-controlled study design, we can now report with confidence that the Intibia System confers a greater QoL impact than placebo.

Historically, randomized sham-controlled studies with tibial nerve stimulation (TNS) have been limited owing to difficulties in conducting a blinded trial. Stimulation of the tibial nerve can often be felt by the patient, limiting the effectiveness of the blinding. In this study, subjects in both groups were informed that they may or may not feel stimulation regardless of receiving active therapy or not. The study’s rigorous commitment to maintaining the blind also specified that subjects were not allowed to have programming adjustments after the 2-week visit and before the 3-month visit. After the 3-month visit, subjects could freely request increases in stimulation to further improve the therapeutic effect, and 80.9% requested this increase in stimulation, underscoring the importance of patient-provider feedback. Although we importantly demonstrate significant improvements in the ITNS-ON group over the ITNS-SHAM group using PGI-I and OABq-SF questionnaires after 3 months of treatment, the further rise in QoL scores at 12 months likely reflects the “real-world” expected QoL improvements with this therapeutic device based on interactive stimulation adjustments.

Previous studies using other methods of TNS have been conducted, with various approaches, to assess superiority over sham controls with QoL and patient satisfaction outcomes. Aydogmus et al. compared a group using transcutaneous TNS device with a sham group with the same self-adhesive electrode placement but without stimulation in patients with OAB. Subjects with active stimulation reported significant improvements in multiple measures of QoL including the ICIQ-UI SF, visual analog scale, and the Overactive Bladder Questionnaire tool V8 compared with the sham group [16]. Peters et al. reported the results of the SUmiT trial: a randomized, double-blind study comparing PTNS with a sham intervention with an altered electrode placement delivering stimulation without the therapeutic effect. After 12 weeks, subjects in the PTNS treatment group reported significant improvements on the OABq compared with the sham group [12]. With this analysis, we now demonstrate a similar significant improvement with the Intibia device.

The QoL and patient satisfaction outcomes reported here support a patient-centered approach, critical to the success of any treatment for UUI [17]. MCIDs for OABq and ICIQ-UI SF scales are important metrics that indicate clinically meaningful, relevant changes to QoL for the patient. In the ITNS-ON group, over 71% and 74% of subjects reported improvements exceeding the MCID for the OABq-SF Symptom Bother and HRQL scales respectively, and 71.5% exceeded the MCID for the ICIQ-UI SF after 12 months of treatment. Thus, the QoL improvements demonstrated with the Intibia System were both clinically and statistically significant.

Comparison of outcomes between ITNS devices remains difficult owing to differences in study design and patient populations [9–11]. In this study, patients with prior treatment of urinary symptoms with nerve stimulation were excluded and subjects were required to abstain from the concomitant use of OAB medications throughout the trial, which is not reflective of typical practice. Coupled with the double-blind, sham-controlled design, the non-enriched patient population included in the Intibia Pivotal Study contrasts with other pivotal trials in the ITNS space, which allowed for concomitant OAB medication use [9, 11], prior treatment with PTNS or SNS [9–11], or both [9, 11], thereby demonstrating a more isolated effect of the Intibia System on QoL in patients with UUI.

Although this study provides important QoL information from an innovative randomized trial design, there are several notable limitations. First, the overall sample size was calculated based on the primary and secondary outcomes of the pivotal trial and not the additional QoL outcomes reported here. Second, our results may not be generalizable to patients with less severe OAB symptoms, as at least four UUI episodes within 72 h were required for study enrollment. Third, and akin to other ITNS pivotal trials, our study population was predominantly female (98.6%). However, multiple other TNS trials have demonstrated efficacy in mixed cohorts with significant male populations, suggesting similar outcomes [12, 18]. Finally, in order to maintain the integrity of the study design, investigators could not adjust stimulation levels during the sham-controlled portion of the study or offer concomitant drug therapy, which is not reflective of standard clinical practice and may limit the generalizability of the results.

## Conclusion

The Intibia System, an ITNS device, demonstrated significant improvements on patient-reported QoL metrics compared with sham-controls after 3 months of treatment. During the open-label extension of the trial, with the ability to increase stimulation levels to individualized targets, we observed further significant increases in global impression of improvement and disease-specific QoL scales. These results show promise in advancing UUI treatment options.

## Abbreviations

HRQL: Health-Related Quality of Life
ICIQ-UI SF: International Consultation on Incontinence Questionnaire – Urinary Incontinence Short Form
ITNS: Implantable tibial nerve stimulation
ITT: Intent-to-treat
MCID: Minimally clinically important difference
OAB: Overactive bladder
OABq-SF: Overactive Bladder Questionnaire Short Form
PGI-I: Patient Global Impression of Improvement
PTNS: Percutaneous tibial nerve stimulation
QoL: Quality of life
SNS: Sacral nerve stimulation
TNS: Tibial nerve stimulation
UUI: Urge urinary incontinence

## References

[1] D’Ancona C, Haylen B, Oelke M, Abranches-Monteiro L, Arnold E, Goldman H, et al. The International Continence Society (ICS) report on the terminology for adult male lower urinary tract and pelvic floor symptoms and dysfunction. Neurourol Urodyn. 2019;38(2):433–77. 10.1002/nau.23897 30681183

[2] Cameron AP, Chung DE, Dielubanza EJ, Enemchukwu E, Ginsberg DA, Helfand BT, et al. The AUA/SUFU guideline on the diagnosis and treatment of idiopathic overactive bladder. J Urol. 2024;212(1):11–20. 10.1097/JU.0000000000003985 38651651

[3] Stewart WF, Van Rooyen JB, Cundiff GW, Abrams P, Herzog AR, Corey R, et al. Prevalence and burden of overactive bladder in the United States. World J Urol. 2003;20(6):327–36. 10.1007/s00345-002-0301-4 12811491

[4] Coyne KS, Wein A, Nicholson S, Kvasz M, Chen CI, Milsom I. Comorbidities and personal burden of urgency urinary incontinence: a systematic review. Int J Clin Pract. 2013;67(10):1015–33. 10.1111/ijcp.12164 24073974

[5] Sexton CC, Coyne KS, Vats V, Kopp ZS, Irwin DE, Wagner TH. Impact of overactive bladder on work productivity in the United States: results from EpiLUTS. Am J Manag Care. 2009;15(4 Suppl):S98–107. 19355804

[6] Perry S, McGrother CW, Turner K. An investigation of the relationship between anxiety and depression and urge incontinence in women: development of a psychological model. Br J Health Psychol. 2006;11(Pt 3):463–82. 10.1348/135910705X60742 16870056

[7] Tutolo M, Ammirati E, Heesakkers J, Kessler TM, Peters KM, Rashid T, et al. Efficacy and safety of sacral and percutaneous tibial neuromodulation in non-neurogenic lower urinary tract dysfunction and chronic pelvic pain: a systematic review of the literature. Eur Urol. 2018;73(3):406–18. 10.1016/j.eururo.2017.11.002 29336927

[8] Du C, Berg W, Siegal AR, Huang Z, Jeong R, Hwang K, et al. Real-world compliance with percutaneous tibial nerve stimulation maintenance therapy in an American population. Urology. 2021;153:119–23. 10.1016/j.urology.2021.02.005 33581232

[9] Rogers A, Bragg S, Ferrante K, Thenuwara C, Peterson DK. Pivotal study of leadless tibial nerve stimulation with eCoin® for urgency urinary incontinence: an open-label, single arm trial. J Urol. 2021;206(2):399–408. 10.1097/JU.0000000000001733 33797291

[10] Lee U, Xavier K, Carey J, Pickering A, Harris-Hicks J, Giusto L, et al. Implantable tibial neuromodulation therapy improves symptoms of urge urinary incontinence from the TITAN 2 pivotal study. J Urol. 2026. 10.1097/JU.0000000000004958. PMC13218429 41587348

[11] Heesakkers J, Toozs-Hobson P, Sutherland SE, Digesu A, Amundsen CL, McCrery RJ, et al. A prospective study to assess the effectiveness and safety of the BlueWind System in the treatment of patients diagnosed with urgency urinary incontinence. Neurourol Urodyn. 2024;43(7):1491–503. 10.1002/nau.25477 38634481

[12] Peters KM, Carrico DJ, Perez-Marrero RA, Khan AU, Wooldridge LS, Davis GL, et al. Randomized trial of percutaneous tibial nerve stimulation versus sham efficacy in the treatment of overactive bladder syndrome: results from the SUmiT trial. J Urol. 2010;183(4):1438–43. 10.1016/j.juro.2009.12.036 20171677

[13] Zapico Á, Ercilla J, Angulo JC, Pérez V, Cuenca JN, Barreira-Hernández D, et al. Effect of neuro-adaptive electrostimulation therapy versus sham for refractory urge urinary incontinence due to overactive bladder: a randomized single-blinded trial. Journal of Clinical Medicine. 2023;12(3):759. 10.3390/jcm12030759. PMC9917375 36769409

[14] Coyne KS, Matza LS, Thompson CL, Kopp ZS, Khullar V. Determining the importance of change in the overactive bladder questionnaire. J Urol. 2006;176(2):627–32. 10.1016/j.juro.2006.03.088 16813906

[15] Nyström E, Asklund I, Lindam A, Samuelsson E. Minimum important difference of the ICIQ-UI SF score after self-management of urinary incontinence. BMC Womens Health. 2024;24(1):118. 10.1186/s12905-024-02947-x PMC10865549 38355503

[16] Aydogmus H, Sengul M, Bolel O, Kotan TS. Effectiveness of transcutaneous tibial nerve stimulation on overactive bladder treatment. Pak J Med Sci. 2025;41(6):1629–37. 10.12669/pjms.41.6.11658 PMC12223738 40621528

[17] Nolazco JI. Future of urology: Embracing patient-centered care and patient-reported outcomes. Curr Urol. 2024;18(1):23. 10.1097/CU9.0000000000000198 PMC10946648 38505152

[18] Jiang JF, Leung AK, Pettitt-Schieber B, Nabavizadeh R, Thomas RL, Brown M, et al. Efficacy of percutaneous tibial nerve stimulation for overactive bladder in women and men at a safety net hospital. J Urol. 2020;203(2):385–91. 10.1097/JU.0000000000000539 31518202

